# Alpha Synuclein Toxicity and Non-Motor Parkinson’s

**DOI:** 10.3390/cells13151265

**Published:** 2024-07-27

**Authors:** Gabriella M. Mazzotta, Carmela Conte

**Affiliations:** 1 Department of Biology, University of Padova, 35131 Padova, Italy; 2 Department of Pharmaceutical Sciences, University of Perugia, 06126 Perugia, Italy

**Keywords:** alpha synuclein, Parkinson’s disease, non-motor symptoms, early diagnosis

## Abstract

Parkinson’s disease (PD) is a common multisystem neurodegenerative disorder affecting 1% of the population over the age of 60 years. The main neuropathological features of PD are the loss of dopaminergic neurons in the substantia nigra pars compacta (SNpc) and the presence of alpha synuclein (αSyn)-rich Lewy bodies both manifesting with classical motor signs. αSyn has emerged as a key protein in PD pathology as it can spread through synaptic networks to reach several anatomical regions of the body contributing to the appearance of non-motor symptoms (NMS) considered prevalent among individuals prior to PD diagnosis and persisting throughout the patient’s life. NMS mainly includes loss of taste and smell, constipation, psychiatric disorders, dementia, impaired rapid eye movement (REM) sleep, urogenital dysfunction, and cardiovascular impairment. This review summarizes the more recent findings on the impact of αSyn deposits on several prodromal NMS and emphasizes the importance of early detection of αSyn toxic species in biofluids and peripheral biopsies as prospective biomarkers in PD.

## 1. Introduction

Parkinson’s disease (PD) is one of the most common neurodegenerative disorders, second only to Alzheimer’s disease, and refers to α-synucleinopathies characterized by loss of the dopaminergic and monoaminergic neurons in *substantia nigra pars compacta* (SN*pc*) and ventral tegmental area, as well as by the accumulation, aggregation and spread of α-Synuclein (αSyn) within neurons and non-neuronal cells including microglia, pericytes, astrocytes, and oligodendrocytes [1]. PD is the most common form of movement disorder clinically manifested by bradykinesia, resting tremor, rigidity, slowness of movement, freezing, dizziness, and postural instability as a result of the loss of 70–80% of the dopaminergic neurons in the SN*pc* [2]. Aside from motor manifestations, PD impacts a plethora of non-motor symptoms (NMS) which may predate the onset of movement disorders by several years [3,4]. They include alterations in the sense of smell [5,6], dysfunction of circadian rhythms [7], and autonomic disorders such as orthostatic hypotension constipation, urinary difficulties, gastrointestinal disorders, anxiety, depression, and impairment or loss of sensory perception [4,8,9]. Non-motor manifestations have been recognized as significant contributors to the overall impact of the disease on the individual’s quality of life [10,11,12].

According to disease stagings by Braak, in stage 1, early NMS such as smell disorders experienced by PD patients are caused by neuronal degeneration in the olfactory bulb and the anterior olfactory nucleus, while degeneration in Braak stage 2 involves the lower brainstem i.e., raphe nucleus, locus coeruleus (LC), pedunculopontine nucleus, and the thalamocortical system, which are associated with sleep disturbances, autonomic dysfunctions, visual hallucinations, and rapid eye movements [13].

Dysfunction of the medullary nuclei located in the brainstem, namely nucleus tractus solitarius, dorsal motor nucleus of the vagus, and nucleus ambigus, regulate various involuntary functions including arterial blood pressure, heart rate, respiratory activity, and renal function. These conditions are associated with orthostatic hypotension, cardiovascular abnormalities, and other autonomic disorders in PD [14]. The emergence of the classic motor symptoms of PD typically occurs at Braak stages 3 and 4. These clinical signs coincide with the involvement of key regions such as the SN*pc* and other deep nuclei of the midbrain and the forebrain [15]. In the final stages Braak 5 and 6, Lewy bodies are found in the limbic structures and mature neocortex. At these stages, patients with PD experience a variety of neuropsychiatric symptoms, including depression, cognitive impairment, and visual hallucinations [15]. The subthalamic nucleus plays a crucial role in PD. It receives inputs from sensorimotor, associative, and limbic brain regions which serve distinct functions within the basal ganglia circuitry. Specifically, pallidosubthalamic projections are thought to underlie cognitive, emotional, and motivational NMS. This intricate organization underscores the diverse roles of the subthalamic nucleus in both motor and non-motor aspects of PD.

Several studies have reported the positive effect of deep brain stimulation (STN-DBS) on motor and NMS, although PD patients with longer disease duration had limited motor benefits from STN-DBS [16,17,18,19]. Case reports and data from a meta-analysis conducted on dozens of studies indicate that STN-DBS improves NMS and PD patients’ quality of life [20,21,22,23]. Due to the complexity of PD, a more comprehensive understanding of the underlying mechanisms beyond the scope of the Braak staging model is required. Certainly, the recognition of non-motor signs that include other issues such as cognitive and memory impairment known as pre-motor symptoms, can contribute to early diagnosis of PD and help adopt strategies aimed at improving the management of the disease [24,25]. In this review, we highlight recent advancements in the field of αSyn neurotoxicity that have contributed to our understanding of NMS onset that could aid in the development of future treatment strategies and/or early diagnosis.

## 2. αSyn Toxicity

From a pathological point of view, similar to motor symptoms, NMS can occur as a consequence of the formation of intracytoplasmic Lewy bodies and neurites rich in αSyn aggregates in nigral and extranigral areas.

αSyn is a small, 140 amino acid presynaptic acidic protein encoded by the *SNCA* gene whose main function appears to be the control of neurotransmitter release [26]. It is a vesicle-bound multimer consisting of three distinct regions: (1) an N-terminus (residues 1–60) region that is crucial for its interaction with lipid membranes [27,28]; (2) a central hydrophobic region (residues 61–95) that is prone to aggregation and named the non-amyloid-β component (NAC region) [29]; (3) an unstructured C-terminus (residues 96–140) that is highly negatively charged with Ca^2+^ binding and chaperone-like activity [30].

In the CNS, αSyn exists in a soluble cytosolic fraction (for as much as 1% of the total protein) and two membrane- and vesicle-binding forms: via the C-terminal domain it interacts with the vesicle-associated membrane protein 2 (VAMP2) leading to the formation of vesicle clusters [31] and affecting vesicle docking as well as the inhibition of vesicle fusion, while via the N-terminal region αSyn was found to bind membrane lipids. This membrane-binding pool appears to prevent pathological aggregation of αSyn [32,33,34]. Mutations in this domain are associated with PD pathology [35,36]. On the contrary, the cytosolic fraction is intrinsically disordered and behaves like a natively unfolded protein, contributing to the formation of aggregate species [37]. αSyn aggregates have been detected in various peripheral biopsies such as the gastrointestinal tract, skin, and salivary glands, even in the early stages of PD [38] supporting the hypothesis that PD might start in the peripheral nervous system before affecting the brain. For instance, αSyn pathology has been found in the enteric nervous system (ENS), leading to the theory that the disease may originate in the gut and then spread to the brain via the vagus nerve [39].

Different factors, such as genetic mutations [40], elevated levels of αSyn, mitochondrial dysfunction [41], oxidative stress (OS) [42], endoplasmic reticulum (ER) stress [43] dysregulation of synaptic vesicle recycling [44], and the autophagy-lysosomal system [45], are well known to contribute to αSyn misfolding, forming β-sheet rich oligomers and fibrils in Lewy bodies or Lewy neurites accompanying NMS [46,47,48,49]. Studies also support the contribution of neuroinflammation in NMS [50,51,52], pointing to the essential components of the innate immune response such as the toll-like receptors (TLRs) [53,54,55,56]. In particular, pathogen-associated molecular patterns (PAMPs) and damage-associated molecular patterns (DAMPs) can prime and activate the TLRs creating a neuroinflammatory status that may culminate with neuronal death in specific brain areas [57]. Human studies suggest a role for TLR2 and TLR4 in the recognition of toxic species of αSyn as a DAMP that precedes αSyn aggregation [58,59]. Furthermore, polymorphisms in the *SNCA* gene are also well-recognized factors contributing to the development of non-motor signs [60]. NMS also result from the diffusion of the pathological species of αSyn. In fact, αSyn aggregates spread between interconnected brain areas in a cell-to-cell and prion-like manner which involves neurons and non-neuronal cells [61,62]. Transsynaptic transmission of αSyn may be triggered by oligomeric αSyn-mediated microglial activation in the early phases of the disease [63] or by interactions between endogenous αSyn and mitochondria [64,65]. However, cells differentially mediate the uptake of αSyn fibrils involving processes such as receptor-mediated endocytosis, extracellular vesicles, and tunneling nanotubes. Fibrils can be sequestered and degraded into lysosomes or be trafficked into the cytosol via endocytic pathways, where they can interact with and recruit monomeric αSyns into mature pathological inclusions [66]. In addition, fibrils can be released by damaged or dead cells following mitochondrial dysfunction [41,54,67], disruption of redox balance [42], nitrosative stress [68], impaired autophagic flux [45], and prolonged ER stress [43]. Oligomeric αSyn may alter the voltage-gated receptors resulting in impairment of calcium efflux [69,70].

**Figure 1:**
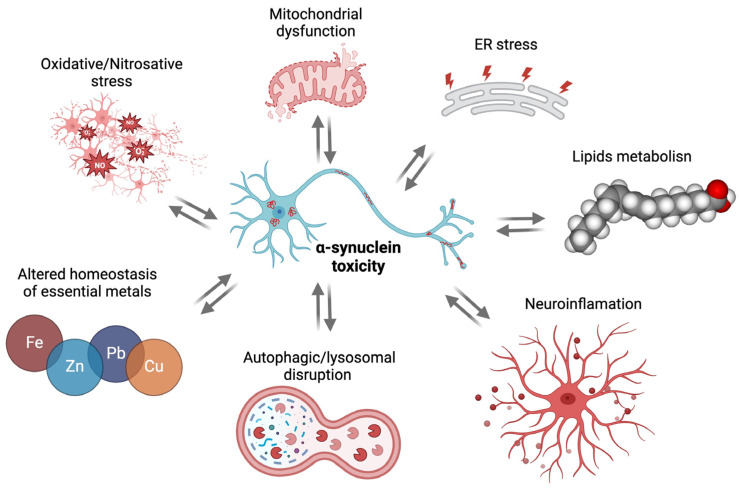
Bidirectional relationship between toxic αSyn species and cellular dysfunctions. Different factors are well known to contribute to the generation of misfolded toxic αSyn species. Additionally, abnormal levels or misfolded forms of αSyn impact several aspects of neuronal function, thus contributing to the pathogenesis of PD. Created with Biorender.com.

A schematic representation of the relation between toxic αSyn species and cellular dysfunctions is illustrated in Figure 1.

### 2.1. Oxidative/Nitrosative Stress

A bidirectional relationship between oxidative/nitrosative stress and αSyn toxicity exists [71]. High levels of reactive oxidative species (ROS) within the neurons have been suggested to induce αSyn toxicity [72,73,74]. Excessive OS causes peroxidation of the membrane lipids followed by the production of the highly toxic 4-hydroxy-2-nonenal (4-HNE). This compound induces the formation of β-sheets and toxic soluble oligomers of αSyn which are believed to promote neuronal damage [75,76]. Hydroxyl radical species can react with tyrosine residues on αSyn contributing to the formation of intramolecular oxidative covalent cross-linkage between two tyrosine residues known as the dityrosine bond [77]. Two opposing effects have been described for dityrosine: (i) formation and stabilization of αSyn insoluble fibrils or aggregates [78], and (ii) inhibition of αSyn fibrillation by dityrosine-modified monomers and dimers [77]. As aggregation proceeds, dityrosine formation shifts from an aggregation-inhibiting to an aggregation-promoting element [79]. The higher-order assembly as well as the increased stability of growing fibrils appears to significantly reduce their capacity to seed further due to the reduced availability of nucleation sites and the conversion to less dynamic structures. Again, changes in hydrophobicity or charge distribution aside from changing the equilibrium between monomers, oligomers, and fibrils could influence the incorporation of additional monomers and aggregation kinetics [80].

Hypochlorite-oxidized cysteinyl-dopamine is considered a potent redox cycler capable of accelerating OS and contributes to increased autophagy and ultimately to cell death [81]. Additionally, post-translationally aberrant S-nitrosylation caused by reactive nitrogen species and nitric oxide in neurons is known to induce αSyn misfolding and toxicity, and axo-dendritic and dopamine dysfunction [82]. Moreover, Kumar et al. [83] showed that S-nitrosylation of the ubiquitin C-terminal hydrolase-1 induces nucleation with the native αSyn, accelerating the protein aggregation. 

### 2.2. Mitochondrial Dysfunction

Mitochondrial dysfunction plays a central role in the pathogenesis of PD [84]. Several neurotoxins have been found to provoke high levels of ROS, complex I inhibition, and neuronal damage [73]. Additionally, abnormal levels or misfolded forms of αSyn can disrupt the balance of mitochondrial fission, fusion and transport, leading to fragmentated or aggregated mitochondria, and impairment of mitochondrial trafficking, electron transport chain, and calcium signaling [84,85]. αSyn can interact with complex I, resulting in decreased ATP production and increased ROS [86]. The selective autophagic degradation of damaged mitochondria can overwhelm cellular proteostasis, leading to the accumulation of misfolded αSyn and mitochondrial dysfunction [87,88,89]. αSyn can trigger the opening of mitochondrial permeability transition pores, causing mitochondrial swelling and loss of membrane potential [90], and can interact with other proteins involved in mitochondrial quality control, such as Parkin and PINK [91]. Dysregulation of these interactions can impair mitochondrial function and dynamics, contributing to the pathogenesis of PD.

### 2.3. ER Stress 

The accumulation of newly synthesized or improperly folded proteins in the ER can saturate the folding machinery, leading to ER stress which promptly activates the unfolded protein response (UPR) to restore normal ER function. In response to prolonged stress, UPR loses the capacity to assist in protein folding and its dysregulation culminates with neuronal death as a protective measure to prevent further damage [43]. In this condition, the activation of C/EBP Homologous Protein by Protein Kinase R-like ER Kinase, Activating Transcription Factor 4 (ATF4), ATF6, or X-box Binding Protein 1 appears to be central for the induction of ER stress-driven apoptotic signaling, ensuring that cells with irreparable damage are eliminated to maintain tissue homeostasis. ER-phagy, a selective form of autophagy targeting the ER for degradation, plays a crucial role in the clearance of misfolded αSyn. The interaction with ER-resident autophagy receptor FAM134B and the involvement of calnexin are key components for the recruitment of unfolded αSyn, encapsulation of ER fragments into autophagosomes, transport to and fusion with lysosomes, and degradation and clearance of misfolded synuclein [92].

### 2.4. Neuroinflammation 

A PubMed search for “inflammation and non-motor symptoms of PD” yielded 302 results from the past 10 years, indicating that the issue is of great interest and importance. Although some debate still ensues, several studies support the crucial role of the immune/inflammatory response in the pathogenesis of NMS in PD. Once triggered, inflammatory load commences a series of toxic events that correlate with a number of NMS including depression, cognitive impairment, psychosis, sleep disturbance, GI dysfunction, and others. The major result is a massive release by microglia, the CNS-resident immune cell of several pro-inflammatory cytokines including IL-1, IL6, TNFα, and IL-17. This may be the result of the activation of a variety of signaling pathways or represent the trigger for NMS, or both. 

The switching of microglia from the M1 to M2 phenotype can be facilitated by endogenous or extracellular αSyn. For example, iPSC-derived macrophages from patients with SNCA triplication display accumulation of αSyn, impairment of phagocytosis, and reduced ability of dopaminergic and GABAergic differentiation [93,94]. On the other hand, extracellular αSyn released by damaged neurons can propagate to neighboring microglial cells and activate microglia via the TLRs-NLRP3 or NF-kB-NLRP3 axes to produce further inflammatory mediators, including caspase-1-mediated IL-1β. αSyn can act as an NLRP3 inflammasome inductor leading to the processing and secretion of mature IL-1β contributing to Lewy body formation in brain tissue of PD patients and in other peripheral tissues. αSyn can stimulate endogenous protein aggregation leading to the spread of αSyn pathology and to the development of NMS and motor symptoms [95]. 

Another important factor associated with the NLRP3 inflammasome is the αSyn-mediated upregulation of the Kv1.3 channel in microglia and the consequent increase in K^+^ efflux. This process could affect dopamine release by neurons, which in turn could inhibit NLRP3 inflammasome formation, constricting the microglia to utilize DA from neighboring neurons to prevent its own activation and thereby avoid the establishment of an inflammatory state that would lead to further αSyn aggregation [96,97]. 

It has been found that the activity of indoleamine-2,3-dioxygenase (IDO), the rate-limiting enzymes of tryptophan metabolism, is induced by high levels of pro-inflammatory cytokines which may lead to decreased serotonin production, depression, and Schizophrenia [98,99]. The treatment of the 6-hydroxydopamine mouse model of PD with IDO inhibitors rescued oxidative stress, neuroinflammation, apoptosis, and dopamine depletion [100]. 

Persistent striatal neuroinflammation is associated with sleep disturbances, cognitive deficits and behavioral changes [101] and may correlate with uncorrected nutritional status [102] or stress accompanying hypothalamic–pituitary–adrenal axis disruption [103]. 

The central role of neuroinflammation in PD includes its ability to impair mitochondrial and lysosome activity via dysregulating αSyn folding and clearance. Mitochondrial dysfunction is also connected to MAO-B activity in astrocytes [104]. 

Mounting evidence also shows that PD patients exhibit gut inflammation. The molecular mechanisms linking inflammation to αSyn pathology have been described in the “Gastrointestinal symptoms” paragraph of the present review.

Both neurons and glia can release adenosine which when acting on purinergic neurons leads to the secretion of pro-inflammatory cytokines, and the activation of microglia.

These findings raise intriguing questions about the role of neuroinflammation in NMS in PD, which although still questioned, requires further investigations before the development of treatments against this multifaceted and omnipresent phenomenon in PD patients.

### 2.5. Lipids

There is increasing evidence that high levels of oligomeric polyunsaturated fatty acids (PUFA’s) play an important role in neuronal toxicity. Notably, lipid peroxidation of PUFAs represents a feature of PD [105]. Specifically, there is a substantial increase in reactive aldehyde species such as malondialdehyde, 4-hydroxy-2-nonenal, cholesterol lipid hydroperoxide, and F2-isoprostanes in the SN*pc* of patients with Parkinson’s as well as in the anterior cingulate cortex of PD subjects [105,106]. 

Many studies also showed physical interaction between αSyn and PUFAs which results in a higher propensity to pathological aggregation [107,108,109,110]. The mutual electrostatic interaction between αSyn and membranes containing negatively charged lipids is found to affect αSyn’s characteristics and the membrane’s composition. In particular, changes in the levels of fatty acids, sphingolipids, and cholesterol have been reported [111]. 

### 2.6. Autophagic/Lysosomal Disruption

Autophagic/lysosomal disruption is closely linked to αSyn toxicity, particularly in the context of PD. The autophagy-lysosome pathway is a crucial mechanism responsible for the degradation and recycling of damaged or misfolded proteins, which include autophagosome formation, fusion with lysosomes, and degradation by lysosomal hydrolases. Overexpression or aggregation of αSyn can disrupt the autophagy-lysosome pathway leading to the accumulation of misfolded αSyn in the brain, while inefficient clearance of αSyn aggregates facilitates their spread to neighboring cells, propagating proteotoxicity. The translocation of cytosolic αSyn to the lysosomal lumen is mediated by the binding of heat shock cognate 70 chaperone to the KFERQ sequence which, with other co-chaperones, directs αSyn to the chaperone-mediated autophagy (CMA) adaptor Lysosomal-Associated Membrane Protein 2 (LAMP2A) located on the lysosomal membrane. Under stress conditions, exposure of the KFERQ motif can trigger CMA to remove harmful αSyn. Dysregulation of CMA is related to impaired degradation of αSyn and may contribute to the pathogenesis of PD [112].

Autophagy of mitochondria, or mitophagy, is intricately regulated by specific posttranslational modifications that “tag” the cargo for degradation, and by the Tank-binding kinase 1-mediated phosphorylation of *optineurin* that enhances its binding to ubiquitin and light chain 3 (LC3). Once bound to the ubiquitinated mitochondria, adaptors recruit LC3 so that the mitochondria are encapsulated by a double membrane structure called the autophagosome. Then, the autophagosome fuses with a lysosome forming an autolysosome, where the mitochondria are degraded and recycled [113]. Elevated αSyn levels inhibit macrophagic flux. Moreover, mutant forms of αSyn, such as A53T and A30P, exhibit a stronger binding affinity for LAMP2A that impairs the efficiency of αSyn clearance [114]. A53T and E46K αSyn variants also engage functional LC3B monomers into insoluble microaggregates on the surface of late endosomes favoring αSyn exosome excretion and seeding [82].

### 2.7. Metals 

Although the precise role of metals in the pathogenesis of PD is still debated, evidence suggests their involvement in conformational effects is related to the binding of metal ions to αSyn and subsequent aggregation and accumulation. Elevated levels of metals such as iron, zinc, aluminum, lead, and copper have been found in the brain and in the cerebrospinal fluid (CSF) of PD patients [115]. Albeit with different affinities and stoichiometries, metals, especially copper, can establish electrostatic interactions with the C-terminus of αSyn, and more strongly phosphorylate Tyr-125 and Ser-129 of αSyn (p-αSyn), increasing their propensity to fibrillation. The aggregation speed of the acetylated A53T variant is higher with respect to the wild-type protein, suggesting an intrinsic self-assembly of the αSyn mutants into aggregates [116]. The toxicity of iron has also been investigated. Abeyawardhane et al. [117] suggest that the impact of Fe(II) on αSyn structure is higher than Fe(III) because of its elevated reactivity with O_2_, resulting in the production of H_2_O_2_ and triggering β-sheet generation; it can act both as an initiator and as a potential allosteric cofactor of protein misfolding [118]. The interaction of αSyn with both oxidation states of copper ions (Cu(I) and (Cu(II)) generates ROS, and contributes to OS potentially leading to its aggregation into pathological fibrils [119]. Aluminum is a metal that can cross the blood-brain barrier (BBB) and accumulate in the brain [120]. It has been found to co-localize with Biondi ring tangles in PD brains [121]. There is evidence suggesting that aluminum can induce OS, directly interact with αSyn, and disrupt the balance of essential ions in the brain, thus accelerating the fibrillation process of αSyn aggregation and contributing to the development and progression of PD.

## 3. Non-Motor Symptoms Associated with αSyn Pathology

**Figure 2:**
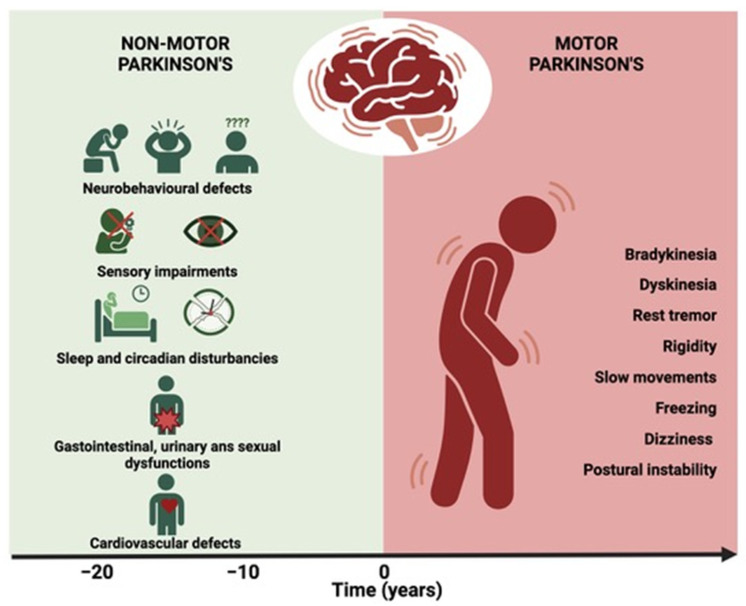
Prodromal non-motor symptoms in synucleinopathies. In the prodromal stage of synucleinopathies, several non-motor symptoms can appear up to 20 years before cardinal motor symptoms manifest. Created with Biorender.com.

Non-motor symptoms in PD include both sympathetic and parasympathetic dysfunctions and may concern, among other issues, neurobehavioral changes, pain, olfaction impairment, sleep and circadian dysfunctions, gastrointestinal symptoms, urogenital disturbances, and cardiovascular problems. Figure 2 illustrates the cardinal non-motor symptoms in the prodromal stage of PD.

### 3.1. Depression

The wide spectrum of NMS implicates the molecular interaction between the dopaminergic, glutamatergic, noradrenergic, and serotoninergic systems in which a significant number of neurons are lost [122]. Depression is a psychiatric condition estimated to affect approximately half of all PD patients [123]. Approximately 17% of individuals recapitulate symptoms consistent with major depression [124,125]. Research indicates that αSyn expression is upregulated in major depressive disorder (MDD) patients and that the pathology begins with the early accumulation in the olfactory system, particularly in the anterior olfactory nucleus, and then spreads to the limbic system. Moreover, neuroimaging studies revealed alterations in the limbic system including the cingulate gyrus, hippocampus, amygdala, hypothalamus, nucleus accumbens, ventral striatum, and orbitofrontal cortex [126]. Unfortunately, L-DOPA, the gold standard for the treatment of motor symptoms, does not alleviate depressive symptoms, indicating that depression is associated with deficits in serotoninergic neurotransmission in the brainstem, raphe nuclei, and limbic system circuitry [127,128]. In fact, serotonin and its metabolite, 5-HIAA, is decreased in the plasma of PD patients with depression [129]. Although the precise mechanisms for depression in PD are not fully elucidated, growing data reports the presence of aggregates of αSyn in the monoaminergic brainstem structure, specifically in dendritic and axonal processes that, associate with the loss of neurons in the aforementioned brain areas, giving rise to disturbances of neuroplasticity and depressive signs that occur in PD [130,131,132]. A recent study showed that adeno-associated virus (AAV5)-induced overexpression of wild-type human αSyn in r 5-HT neurons in the raphe nucleus causes progressive accumulation, phosphorylation, and aggregation of αSyn, an increases in brain-derived neurotrophic factor deficiency (BDNF), and alteration of 5-HT and norepinephrine (NE) systems, all affecting mood control [133]. Moreover, dysregulation of NE transmission may contribute to chronic neuroinflammation. Transgenic mice expressing human αSyn developed toxic species of αSyn in LC neurons, upregulation of GFAP with astrogliosis, microglial abundance, neuroinflammation, LC fiber degeneration, disruption of DA metabolism, and dysregulated NE neurotransmission, which results in changes of emotion and appearance of depressive symptoms [132,133]. Overexpression of αSyn in the hippocampus potently reduced the levels of the SNARE proteins Syntaxin, synapsin 1/2, and Vamp2 and activated C1qa, C1qb, C1qc, C4a, C4b, and C3 complement components, inducing gliosis and increasing hippocampal levels of IL-1b, IL6, and TNFα cytokines. Furthermore, the microglial phagocytosis marker CD68, lysosome marker Lamp1, and cell death markers Caspase-3 and Bax, were significantly increased in αSyn injected mice. These studies demonstrated that accumulation of αSyn in the hippocampus leads to the impairment of neurogenesis, synapse loss, and neuron death [130].

### 3.2. Anxiety

A high proportion of patients with PD (22.2–66.7%) display signs of anxiety, with females being at higher risk than male patients [134,135,136]. Several forms of anxiety are described in PD, including generalized anxiety, panic attacks, social phobia, agoraphobia, and obsessive-compulsive disorder. Genetic and epigenetic factors may be implicated in anxiety in PD patients. One study has suggested the involvement of gene variants of αSyn as well as LRRK2, DJ-1, PINK1, GBA, and BDNF [137]. Anxiety has been associated with genomic DNA levels of mutant αSyn in a transgenic mouse model overexpressing the human A53T αSyn [138]. Also, serotoninergic dysfunction was observed in an A53T αSyn mouse model [139].

Stoyka et al. [140] showed that intrastriatal injections of fibrils of αSyn caused abnormal accumulation of αSyn aggregates in the cortex, amygdala and pSer129-αSyn inclusions that are resistant to proteinase K in the cortex. Likewise, injection of αSyn preformed fibrils into the bilateral olfactory bulb of A53T transgenic mice spreads into connected regions provoking severe pathology in the hippocampus, bed nucleus of the stria terminalis, and central nucleus of the amygdala with a significant increase in total αSyn and p-αSyn. Animals exhibited hyposmia, anxiety-like behavior, and memory impairment, which are consequences of atrophy, neuronal loss, microglial activation and reactive astrogliosis [141]. These changes contribute to psychiatric disturbances. Miquel-Rio et al. [133] showed that overexpression of human αSyn in raphe 5-HT neurons leads to αSyn accumulation/aggregation in raphe nuclei and anxiety-like phenotypes. 

Increase in hippocampal αSyn expression has also been detected in rats with high levels of innate anxiety, suggesting that anxiety can cause αSyn accumulation, in addition to being a contributor of synuclein progression [136]. Importantly, the vulnerability to forming inclusions is higher in glutamatergic excitatory neurons compared with inhibitory neurons. Alteration of calcium homeostasis and lower expression of proteins involved in the αSyn degradation machinery could accelerate the formation of aggregates [142,143].

The symptoms of anxiety, as well as depression and apathy in PD can also be caused by a marked loss of serotonergic neurons compared to dopaminergic neurons in the caudate nucleus, hypothalamus, and frontal cortex [144]. In addition, elevated levels of NE in plasma and CSF have been found in PD patients [145].

### 3.3. Psychosis

Psychosis is a premotor neuropsychiatric condition affecting PD patients [146], characterized by a spectrum of mental symptoms including illusions, delusions, visual hallucinations, schizophrenia (SCZ), and rapid eye movement sleep behavior disorder that are the result of dopamine hyperactivity in the mesolimbic pathway [147,148] and dysfunction of neurotransmitter systems [149]. In the past, psychotic individuals such as schizophrenics treated with antidopaminergic and neuroleptic drugs manifested parkinsonism symptoms. The use of newer atypical antipsychotic drugs has significantly reduced the incidence of psychiatric symptoms [150]. On the other hand, PD psychosis (PDP) is a common side effect derived from dopamine used to treat motor symptoms. However, some data indicates that psychosis may predate motor signs even in the absence of therapy as well as in PD patients, and when manifested is associated with high mortality and morbidity and heavily impacts the quality of life of many patients. There is controversial evidence regarding the role of αSyn in the pathophysiology of PDP. For example, studies have reported downregulation of αSyn expression [151] and lower levels of serum αSyn in SCZ patients compared to healthy controls [152] while another study failed to identify significant differences between SCZ patients and healthy controls [153]. A case study reported that duplication of the *SNCA* gene was associated with the progression of PD [154]. Additionally, αSyn aggregates could contribute to neuroinflammation, OS, synaptic dysfunction, and downstream neurotransmitter imbalance, the main features of schizophrenia [155]. Furthermore, a pilot study revealed the association between the rs356219 polymorphism in the *SNCA* gene and psychiatric disorders compared to healthy controls [156]. Data from a human study described αSyn deposition in specific regions involved in directing attention toward visual targets eliciting hallucination in dementia with Lewy bodies [157]. Furthermore, transgenic rats overexpressing human αSyn (αSyn-BAC, harboring the full-length human *SNCA* locus) showed elevated levels of dopamine as a compensatory response, that was accompanied by aberrant αSyn formation such as hyperphosphorylation, and monomeric C-terminal truncation followed by aggregation pathology. These alterations produced a psychosis-like phenotype [148]. A recent study supports the hypothesis that aberrant lysosome enzyme activities and sphingolipid metabolism may be responsible for αSyn accumulation and SCZ. In particular, decreases in acid sphingomyelinase and increases in alpha-galactosidase activities and αSyn levels were observed in late-onset SCZ patients in comparison to controls [158]. Moreover, an inverse correlation between apolipoprotein E and αSyn was described in PD SCZ [159]. An impaired ubiquitin/proteasome system and mitochondrial dysfunction could contribute to the reduction of the striatal cytochrome oxidase, energy metabolism, and glucose utilization in SCZ [160]. Alteration of the STAT3/mTORC2 signaling pathways in the hypothalamus of Thy1-αSyn transgenic mice seems to associate with the disruption of feeding behavior and energy metabolism and interfere with αSyn-linked pathology and SCZ phenotypes [161].

### 3.4. Cognitive Impairment 

Cognitive decline is a prominent feature of PD and dementia with Lewy bodies (DLB) which comprises deficits ranging from mild cognitive impairment to severe PD dementia (PDD). 

It is not fully understood whether αSyn directly impacts cognition and cognitive progression in PD. However, studies have mainly focused on changes in αSyn levels, properties, and function. High levels as well as low levels of total αSyn in the CSF are found to worsen cognitive performance [162,163]. A prospective study in PD patients and controls demonstrated that low levels of Aβ42 in the CSF were identified as an αSyn-independent predictor of cognitive decline in PD. Aβ_42_ is a peptide that is associated with Alzheimer’s disease, the most prevalent form of dementia. Nevertheless, high plasma αSyn levels have recently been associated with a higher prevalence of cognitive decline and faster progression of clinical dementia [164]. Likewise, LB pathology, evaluated by a seed amplification assay [165,166,167,168] is associated with increased progression of DLB and rapid early alterations in cognitive performance [169]. These findings suggest a transport of CNS αSyn to the periphery that could help clinicians detect, by non-invasive practices, the αSyn levels as early as possible serving as a potential surrogate biomarker of risk of cognitive impairment. A recent transcriptomic study in an αSyn-based model of PD showed region-specific gene expression changes associated with cognitive deficit [170]. 

Mutations in the αSyn gene, including A53T, E46K, H50Q, G51D, A53E/A53V, and αSyn triplication are correlated with cognitive decline [171]. The interplay between LRRK2 and αSyn phosphorylation was demonstrated in postmortem PD patients [156]. Additionally, Rab GTPases are substrates of LRRK2, and its phosphorylation positively regulates αSyn aggregation [156].

Many microRNA including miR-7, miR-129, miR-135a, miR-153 have been recognized to post-transcriptionally regulate αSyn and thereby associated with cognitive decline [172]. 

Importantly, αSyn in the plasma, urine, CSF and other biofluids, can be derived from lipid bilayer-encapsulated particles secreted by cells, known as extracellular vesicles, that reflect the internal status of neurons. High levels of EV αSyn have been found in Alzheimer’s and PD patients and their presence has been associated with the prion-like propagation of αSyn aggregates. 

Lysosome dysfunction stimulates the release of αSyn-containing vesicles in recipient cells, and although the precise role remains to be elucidated, ubiquitination and sumoylation have been proposed as possible mechanisms for αSyn exosome secretion, especially in condition of impaired autophagy [173].

Furthermore, it has been shown that the β-glucocerebrosidase substrate, glucosylceramide, may affect the stabilization of soluble oligomeric intermediates of αSyn accelerating the self-propagation of disease and inducing dementia. In turn, αSyn inclusions may inhibit β-glucocerebrosidase activity by interfering with ER-Golgi trafficking and thereby propagating further accumulation of αSyn [174]. It is important to note that tau filaments and αSyn synergistically promote fibrillation contributing to the development of cognitive impairment in PD patients [175]. The levels of Aβ plaques and tau tangles are also correlated with cognitive performance in patients with PDD suggesting the coexistence of amyloid β and tau pathology [176].

Aside from αSyn accumulation, oxidative stress, astrocyte-associated inflammation and astrogliosis occurring in brain regions including the frontal cortex and hippocampus can promote cognitive dysfunction in PD. Pyramidal cells in hippocampal fields CA3 and granule cells of the dentate gyrus as well as neurons within the amygdala are vulnerable to oxidative stress with negative functional consequences on dendritic growth and neuronal connectivity. 

Several mutations of astrocytic TAR DNA binding protein (TDP-43) in the hippocampus have been shown to increase oxidative stress, mitochondrial dysfunction and lipid peroxidation [177] further contributing to the αSyn neurotoxicity and progressive memory deficit in PD [178].

The improvement of the diagnostic and prognostic roles of αSyn in cognitive deficit in PD could derive from its validation in large independent cohort studies.

### 3.5. Pain 

Neuropathic and inflammatory pain are common NMS in PD that can develop many years before the onset of classic motor signs and significantly impact the quality of life of patients. These NMS have been reported in approximately 60–70% of patients and can vary in nature and prevalence. According to Ford’s classification, five types of pain are proposed: musculoskeletal pain, radicular-neuropathic pain, dystonic pain, central neuropathic pain, and akathisia [179]. Central pathways involved in PD pain comprise the lateral pain pathways with projections to the thalamus, primary somatosensory cortex, and the medial spinoreticulothalamic pathways intimately associated with the autonomic nervous system, containing fibers that project to the medullary core and mesencephalon. Neuronal loss and Lewy body formation occur in the medial pain pathways, specifically in the parabrachial nucleus coeruleus and the periaqueductal grey [180]. αSyn misfolding and aggregation may originate in peripheral nerves fibers and spread along neuroanatomical sensory connections causing a multitude of painful perceptions [181]. Inflammatory pain in PD is caused by an abundance of immune responses in the afferent nociceptive nerve fibers that result in sympathetic nerve fiber damage and leads to the development of characteristic signs of neuropathic pain. PD patients experiencing pain exhibit changes in plasma levels of inflammatory cytokines such as IL1, IL-6, IL-10, and TNFα that actively participate in the perturbations of the proteostasis network, αSyn misfolding, and αSyn proteotoxicity [182].

In a spared nerve injury (SNI) model of neuropathic pain, the expression of αSyn has been detected in peptidergic and non-peptidergic nociceptive neurons in the dorsal horn of the spinal cord, signifying an involvement in pain transmission in the CNS. In the same model, the inhibition of αSyn was associated with the suppression of the pronociceptive MAP kinase signaling and an increase in inflammatory mediators in the spinal cord, probably consequent to the activation of immune cells [183]. In addition, Chen et al. [184] showed that δ-Opioid receptor activation attenuated MPP(+) and hypoxia induced αSyn overexpression/aggregation by enhancing CREB phosphorylation and TORC1/SIK1/CREB pathways. Yi et al. [185] have found that spinal cord ligation is able to induce increases in PINK expression and aberrant mitophagic flux selectively in GABAergic interneurons that may be related to neuropathic pain in neurodegenerative diseases. Interestingly, deficiency of the TMEM175 neuronal proteins impairs lysosomal and mitochondrial function and increases αSyn aggregation [186]. Overall, these findings highlight that αSyn homeostasis represents a key pathogenic factor in the different types of pain. 

### 3.6. Olfactory Dysfunction 

Olfactory dysfunction (OD) is an early NMS symptom of PD affecting more than 90% of patients whose underlying mechanisms are partly defined. Anosmia, the loss of the sense of smell, and ageusia, the loss of taste, have been described as pre-motor symptoms of PD widely recognized as a very early biomarker of the disease and strongly associated with cognitive function [187].

Post-mortem studies of PD brain indicate that αSyn pathology starts in the OB and lower brainstem and developments in limbic system and connected brain regions leading to hyposmia [188]. 

Animal models created by injecting recombinant preformed αSyn fibrils in the bilateral olfactory bulb (OB) exhibit Lewy Body-like pathology and OD [189,190]. Additionally, the human A30P mutant αSyn-expressing mouse model displays OD along with a reduction in OB neurogenesis and alterations in synaptic vesicular transport [191]. The induction of the αSyn aggregates via overexpression of double mutant human αSyn (A53T and A30P) in the OB, negatively impacts neural activity and odor-evoked response in the OB [192]. A recent study using A53T mutant mice revealed hyperactivity of mitral/tufted cells and disruption in excitation/inhibition balance in that OB that impaired GABAergic transmission and abnormal expression of GABA transporters [193]. A study conducted using ^A53T^-α-synuclein transgenic mice revealed a deficit of odor discrimination and odor detection accompanied by a large loss of cholinergic neurons on the mitral cell layer, a decrease in acetylcholinesterase activity, and an alteration in dopamine neurotransmission [194]. The vicious cycle by which the abnormal aggregation of αSyn eliminated neurons, astrocytes, and microglia causes neuroinflammation that in turn promotes additional αSyn aggregation and deposition, has been considered a possible cause of αSyn accumulation in the OB and cell degeneration. 

Intranasal lipopolysaccharide challenge in mice was able to trigger the activation of microglia and IL-1β release followed by the recruitment of IL-1 receptor type I-dependent signaling, indicating that the neuroinflammatory response may be responsible for αSyn pathology [195]. Finally, contracting viral infection during childhood as well as diseases such as asthma, and allergic rhinitis which predispose to the entry of pathogens via the olfactory route, potentially increases the likelihood of developing PD [196,197]. Many viruses, including influenza A, Coxsackie, herpesvirus, and Epstein Barr, have been implicated as either a direct or indirect cause of PD and considered as a prodromal disease [197]. Respiratory viruses enter the brain via the olfactory bulb or Meissner’s plexus, disrupt the BBB, and activate the innate CNS immune system initiating an inflammatory response that persists many years after following the insult generating severe secondary sequelae, similar to parkinsonism. The induction of chemokine and cytokine genes following infections are mediated by TLR signaling and downstream signaling cascades that culminate with the increase in the amount of monomeric and oligomeric αSyn [198]. The inflammatory response induced by the neuro-protective effects of α-synuclein may contribute to its own aggregation. This is an important issue that deserves further investigation.

### 3.7. Visual Impairment

A variety of visual alterations, including ocular, visuoperceptive, and visuospatial impairments have been associated with PD and are mainly caused by DA depletion and loss of amacrine and inner plexiform cells of the retina. Therefore, eye movement, visual acuity, tritan axis of color perception, recognition of visual stimuli, and spatial relationships between objects are substantially compromised in PD patients [199].

αSyn toxicity was found as Lewy neurites in the retina and optic nerve and oligodendroglial cytoplasmic inclusions in the optic nerve. αSyn aggregates and phospho-Syn positive Lewy body-like neurites have also been described in the retina of PD patients, especially in the ganglion cell layer, the inner nuclear layer, and the inner plexiform layer. These aggregates correlate with retinal neuronal death, reduced retinal DA levels, and motor scores at various stages of the disease [200]. Overexpression of human p-αSyn has been observed in the outer nuclear layer (ONL) of the retina of A53T mice and manifests as structural thinning in the ONL and loss of electroretinography that measures a-wave photoreceptor response [201]. A phosphorylated form of αSyn has also been detected in wholemount human retinal nerve fiber and ganglion cell layers from PD and PDD subjects.

A research study has demonstrated that overexpression of αSyn in the retina led to the formation of 129-phosphorylated αSyn, changes in visual acuity, significant loss of TH-positive amacrine cells, and a decrease in dopaminergic amacrine neurotransmission [202]. Moreover, the intravitreal injection of PFF in mice induces the formation of pS129 αSyn inclusions in the visual cortex, perirhinal and entorhinal cortices, alongside an increase in Iba-1, a known marker of microglia activation in the optic nerve. Therefore, it is reasonable that synuclein pathology may be mediated by optic nerve inflammation or by systemic inflammation that spreads to the optical system [203]. It remains to be clarified whether αSyn impairs the function of different intracellular organelles such as the RE, Golgi apparatus, and the mitochondria or whether organelle dysfunction leads to αSyn toxicity. Although further studies need to be performed, crosstalk between αSyn and mitochondrial disfunction has also been previously described. Amacrine cells are interneurons in the retina that form synapses at their presynaptic endings, called lobular appendages. In PD, αSyn accumulation induces mitochondrial loss in the lobular appendages that are consistent with an energetic failure, loss of connexin 36, and impairment in visual signal transmission [204]. Another study using retinal-pigment-epithelial cells revealed that the accumulation of αSyn in these cells worsens lysosome activity by disrupting the trafficking of lysosomal hydrolases. Reduced ferrinophagy can also occur provoking retinal iron dyshomeostasis and consequent cytotoxicity [205].

Overall, these findings suggest the possibility of detecting αSyn in the retina as a promising non-invasive approach for a prodromic biomarker for PD.

### 3.8. Sleep and Circadian Dysfunctions 

Sleep and circadian dysfunctions are recurrent non-motor symptoms of synucleinopathies, and significantly correlate with poorer quality of life [206].

Among the most common sleep-wake disturbances are rapid eye movement (REM) behavior disorder (RBD), a non-familial sleep disorder characterized by dream enactment behaviors the loss of atonia during REM sleep (REM sleep without atonia or RSWA) [207]. RBD is strictly associated with synucleinopaties [208] to the point that idiopathic/isolated RBD (iRBD), that may precede the onset of motor features by decades, is considered a prodromal form of synucleinopathy and is therefore a highly specific marker for future development of the pathology. Furthermore, αSyn aggregates are considered a biomarker for the development of diagnostic assays. For example, αSyn aggregates in stool samples of iRBD patients can be detected and measured, supporting the diagnosis of prodromal synucleinopathies [209].

Transgenic mice overexpressing human A53T α-syn (SNCA^A53T/-^) in its natively expressed regions exhibited REM sleep without atonia, similar to RBD patients, at 5 months of age [210]. Compared to wild-type mice, in whom REM sleep is characterized by sinusoidal theta-frequency electroencephalographic activity and absent or minimal electromyogram activity, SNCA^A53T/-^ mice displayed excessive muscle twitches in body and limb during REM sleep as well as transient increases in muscle activity [208,211,212].

Restless leg syndrome (RLS), a very common movement disorder manifested by disturbing symptoms in lower limbs, more severe in the evening or night, has a high prevalence in PD patients [213] and some studies report the onset of PD at a younger age in subjects with RLS [214].

Insomnia and excessive daytime sleepiness (EDS) are also common in PD patients. Insomnia has been associated with depressed mood, autonomic symptoms fatigue, and age [215]. Subjective EDS has been reported in up to 50% of patients with PD [212]; it has been associated with the neurodegenerative process itself, that is extended to the dopaminergic and non-dopaminergic neurons in the lower brainstem and midbrain [216] involved in sleep-wake regulation [217]. These observations of poorly consolidated rest/activity patterns in humans are paralleled in animal models. Rodent models for PD display impairment in the sleep-wake parameters, such as deficit in REM sleep, overwhelming episodes of sleep, similar to “sleep attacks”, and increased sleepiness [218,219]. In *Drosophila*, the pan-neuronal overexpression of pre-fibrillar human αSyn oligomers impacts the sleep-like rest behavior (defined as the absence of movement for 5 consecutive minutes), in terms of number and length of sleep episodes and total sleep [220,221,222,223]. Flies exhibit decreased sleep (especially at night), increased number of sleep bouts (indicating sleep fragmentation), and decreased wake activity [220,222,223]. These alterations precede the onset of motor symptoms and can be related to the presence of pre-fibrillar αSyn oligomers that prevent protein aggregation [220,221,223]. 

Sleep is coordinated by the interaction of homeostatic and circadian mechanisms, that regulate sleep debt and the periodicity of sleep/wake propensity, respectively [224]. Circadian rhythms are controlled by an endogenous mechanism that comprises: a pacemaker, orchestrated by interconnected transcriptional/translational feedback loops, input pathways for light and other stimuli that synchronize the pacemaker to the environment and output pathways that convert the molecular oscillation of clock components in overt rhythms [225]. In the primary loop, the molecular oscillation involves the transcription factors CLOCK and BMAL1, which, as a heterodimer, regulates the rhythmic expression of output genes. The CLOCK/BMAL1 complex also activates the expression of the negative elements of the loop, Period, and Cryptochrome genes, whose products inhibit their own expression by inactivating the activity of CLOCK/BMAL1 [225]. The circadian physiology is based on a hierarchical network of central and peripheral oscillators; the central pacemaker (master clock) is located in the suprachiasmatic nuclei (SCN) of the hypothalamus: it receives signals from the environment and transmits temporal information to downstream peripheral clocks, located in organs such as the heart, lungs, liver and adrenal glands, through neurotransmitters and neuromodulators [225]. Melatonin secretion is a rhythmic output generated by the SCN: melatonin and circadian clock gene expression can be easily measured in serum and peripheral blood mononuclear cells, respectively, serving as reliable markers of circadian rhythmicity [225].

Several studies have reported lower levels and dampened melatonin oscillation and a lack of time-dependent variation in BMAL1 expression in PD patients, compared to healthy controls, indicating that the peripheral molecular clock is affected in PD [109,211,226].

Additionally, in Drosophila, the sleep defects reported in flies overexpressing pre-fibrillar human αSyn oligomers are associated with a severe impairment of two key circadian features, the anticipation of the dark/light transition and the circadian periodicity [220]. 

Daily metabolic demand of an appropriate distribution of metabolites within tissues is regulated by circadian rhythm. These metabolites in turn regulate many proteins of the clock machinery [227]. Cutting-edge metabolomic studies could help map the distribution of the metabolome in brain tissues. An interesting study by Peek et al. showed that NAD^+^ metabolite is crucial in regulating the circadian rhythm and also activating the NAD^+^-dependent sirtuin 3 (SIRT3), a mitochondrial protein playing a crucial role in mediating mitochondrial oxidative function and autophagy [228]. It has been found that deposition of αSyn oligomers in mitochondria causes downregulation of SIRT3 activity and a decrease in mitochondrial biogenesis, indicating a crosstalk between αSyn, SIRT3 and circadian rhythms [229].

### 3.9. Gastrointestinal Symptoms

The involvement of αSyn as an important player in the complex process leading to GI symptoms in PD is well documented. Several large cohort and case-control studies show correlations between gastrointestinal (GI) dysfunctions and PD, DLB, and PDD during the disease course and overlap with autonomic symptoms [230,231].

GI disorders have been suggested to both cause PD as well as increase the risk of developing PD. Two distinct subtypes of PD have been initially proposed based on the origin and progression of αSyn pathology: a “brain-first” subtype, in which αSyn pathology originates in the OB or other brainstem regions and then spreads to the peripheral autonomic nervous system, and a “body-first” subtype in which the pathological process originates in the enteric nervous system and then ascends via the vagus nerve or other autonomic pathways to reach the brain [232]. However, recent post-mortem studies showed that Lewy pathology is triggered in the GI tract or autonomic nervous system without concomitant involvement of the OB, therefore supporting a single-hit brain-first hypothesis [233,234].

Loss of enteric dopamine cells and degeneration of vagal nuclei represent the main cause of GI symptoms such as dysphagia, sialorrhea, bloating, nausea, vomiting, gastroparesis, and constipation.

p-αSyn deposits in vagus nerve Schwann cells resulted in inflammatory response induced by interaction and activation of a family of pattern recognition receptors, Toll-like receptor 2 (TLR2), implicated in regulation of αSyn release [235]. On the other hand, the interaction between p-αSyn and TLR2/4 has been previously described in murine models of PD as well as in post-mortem human PD brain and is associated with pro-inflammatory responses and PD pathology [236,237]. p-129Syn was detected in submandibular gland samples in PD patients as well as the distal esophagus, while a progressive decrease was observed from the stomach to the small and large intestine and rectum [238].

GI symptoms affect nearly 80% of subjects before or after the onset of motor manifestations and some of them are life-threatening symptoms, such as dysphagia which increases the risk of pneumonia. Furthermore, PD patients experience a more widespread onset of lifelong GI symptoms, and constipation is widely recognized as one of the most prevalent NMS of PD. The intestinal microbiome also play a role in the bidirectional communication between the brain and the gut has emerged as a significant aspect of PD pathology [239,240,241].

Aberrant aggregation of αSyn is responsible for vagus nerve and ENS degeneration, decreased intestinal peristalsis, and constipation [242,243]. A study revealed that human A53T αSyn transgenic mice exhibit severe signs of GI dysfunction together with αSyn aggregation in the ENS [244]. Interestingly, it has also been shown that pathological species of αSyn can be carried through extracellular vesicles secreted by red blood cells into the GI tract, contributing to the onset and/or progression of this PD pathology.

Gut microbiota of PD patients have been found to be rich in *Enterobacteriaceae* [245]. This microorganism family, natively expresses αSyn, and is able to perform anaerobic nitrate respiration with the production of antioxidant nitrite which initiates a cascade of oxidative events that lead to αSyn aggregation [246].

In recent years, many have speculated about the role of short-chain fatty acids (SCFAs) produced by the gut microbiome, especially butyrate and propionate, on the development of synculeinopathies. The effects of short-chain fatty acids (SCFAs) appear paradoxical and contradictory, likely influenced by factors such as injection routes, mixture composition, and concentration. [247,248,249,250]. For example, lower SCFA levels and higher calprotectin detected in the stool of PD patients correlated with GI symptoms [251]. An interesting preclinical study conducted in an αSyn pre-formed fibrils (αSyn PFFs)-induced rat model of PD documented protective effects of sodium butyrate that reduced inflammatory markers such as TNFα, IL-1, IL-6, and increased DA content [252]. Moreover, fecal transplants from PD patients to αSyn overexpressing mice have been shown to promote αSyn-dependent activation of microglia that was associated with worsening motor deficit, suggesting an important role for gut microbiota in regulating the abnormal aggregation of αSyn, and the involvement of microbiota-gut-brain-axis pathways [253]. For this reason, transplanting probiotics into the gastrointestinal tract of PD patients could be a promising strategy to restore gut dysbiosis, reduce inflammation, and elude αSyn alteration and proteotoxicity.

### 3.10. Sexual and Urinary Dysfunctions

Sexual and urinary dysfunctions are common NMS in PD patients that involve neuronal cytoplasmic αSyn inclusions in the peripheral autonomic nerve fibers. In approximately 70% of patients, nocturia is accompanied by urge urinary incontinence and detrusor hyperactivity, often followed by the appearance of motor symptoms [254,255].

αSyn aggregates and p-αSyn have been found in the frontal cortex, basal ganglia, pontine nuclei, sacral spinal cord, pelvic plexus, and genitourinary tract of PD patients with urinary dysfunction [256]. Significantly lower levels of dopamine transporter uptake, possibly a consequence of degeneration of the nigrostriatal dopaminergic neurons and caudate nucleus, have been found in PD, PDD and DLB patients with lower urinary tract symptoms [257,258,259,260]. An animal study has also revealed the disruption of D1-GABAergic striatal output neurons as a possible cause of bladder hyperactivity in PD, suggesting the complex orchestra behind urinary impairment in PD [261].

Data analysis by employing multidimensional self-administered questionnaires to evaluate sexual performance and relationship dissatisfaction suggests that there is a high prevalence of sexual dysfunction among young-onset male and female PD compared to healthy subjects [262], which is related to severity of depressive symptoms especially in female, motor, psychological, cognitive disturbances [263]. Generally, sexual dysfunction appears after the development of motor symptoms. Tremor, bradykinesia, dyskinesia as well as medication such as dopamine agonists, can interfere with sexual function. However, an elevated number of patients experience sexual dysfunction many years before PD onset. Gender difference in sexual dysfunctions arise from hormones. In fact, a higher protection from DA depletion is provided by the higher exposure to estrogens in females. A number of studies have shown a range of symptoms ranging from hyposexual to hypersexual behaviors. For example, erectile dysfunction has been reported in diagnosed male PD and DLB cases, and loss of libido and orgasmic dysfunction were found in both women and men [264] while hypersexual behaviors are often associated with PD treatment [250]. A study has demonstrated impairment in courtship rituals and in copulation in a Drosophila A30P-mediated PD model [265]. A study in PD patients revealed that sexual dysfunction is more frequent in early-onset PD patients compared to late onset PD patients [266]. The control of sexual functions depends on the projections from multiple areas of the brain to the spinal cord. The parasympathetic system is involved in erectile function and in the release of neurotransmitters such as nitric oxide and acetylcholine. The hypothalamus also plays an important role in controlling sexual performance, contributing to the interaction between nervous and endocrine systems and to the exchange of messages between the brain and the spinal cord.

It is possible that various toxic insults could interrupt or impair this intricate cross-talk inducing, the development of insoluble inclusions, such as αSyn oligomers, and triggering toxic events that lead to neuronal death. Although many other studies need to be performed to identify the molecular mechanisms underlying sexual dysfunctions, genetic factors, such as polymorphisms in the dopamine D4 receptor gene, appear to contribute to individual differences in human sexual behavior [267]. All these studies underline the importance of sexual dysfunction in PD as they negatively impact the quality of life of patients and partnership. A such, it is equally important to address sexual and urinary dysfunction in PD, since symptoms can be treated.

### 3.11. Cardiovascular Symptoms

Epidemiological studies have suggested a possible relationship between the incidence of cardio-cerebrovascular disease and PD, either as risk factors or as manifestations of PD itself [268,269]. Studies using Mendelian randomization revealed that PD is correlated with a high risk of coronary artery disease, stroke, ischemic stroke, and cardioembolic stroke compared with age- and gender-matched healthy individuals [270,271]. On the other hand, the deposition of αSyn in the brain of PD patients may be a potential pathogenic contributor to cardiovascular disease and stroke. The involvement of the cardiovascular system in PD reflects αSyn deposition in sympathetic noradrenergic nerves, defects of the autonomic cardiac innervation and abnormal function of residual noradrenergic endings [272].

The loss of myocardial noradrenergic innervation together with the loss of myocardial norepinephrine caused by Lewy body deposition are responsible for orthostatic hypotension with substantial drop in systolic and diastolic blood pressure of at least 20 mmHg and 10 mmHg, respectively [273]. In PD patients, a significant alteration in the electrophysiological activity and remodeling of the myocardium has also been described. The multi-organ deposition of αSyn in PD extends to myocardial tissue, signifying that αSyn fibrils dispersed in the myocardium may be pathogenic. Myocardial tissues from PD autopsies display p-αSyn deposits and Lewy body pathology that correlate with denervation of sympathetic and noradrenergic endings in cardiac tissue [274,275,276]. Accumulation of αSyn aggregates in paravertebral sympathetic ganglia appear to be chronologically preceded by those in the distal axons of the cardiac sympathetic nervous system, suggesting centripetal degeneration [277]. Of note, cardiac dysfunction can be present even when CNS αSyn deposition is limited to the brainstem, with an absence of clinical symptoms [278,279]. Nigrostriatal dopaminergic neurons are vulnerable to high glucose levels and to alterations in insulin signaling and undergo severe injury producing high levels of ROS, via mitochondrial destruction, and low levels of the antioxidant glutathione. Hyperglycemia associated with high uric acid levels cause inflammation and increase in the risk of cardiovascular disease (CVD) in PD [280].

Additionally, high levels of oxidated low-density lipoproteins (LDL), and LDL-cholesterol, as well as changes in sphingolipid and ceramide metabolism, have been found in PD patients, supporting a higher risk of CVD consequent to a greater propensity to atherosclerotic plaque formation. On the other hand, the interaction between cholesterol and αSyn appears to facilitate αSyn aggregation [281]. It remains unclear whether cardiac α-syn accumulation plays a causative role for orthostatic hypotension in PD people [282].

## 4. Conclusions

Given the growing incidence and the long list of NMS in the prodromic stage of PD, as well as the negative impact on health-related quality of life, it is imperative for clinicians to achieve an early diagnosis aimed at prompt intervention to alleviate the symptoms and hopefully slow the progression of the disease. The correlation between αSyn deposition and the appearance of NMS in the 2- to 20-year pre-motor deficit period is well-documented, although the exact mechanisms have not been fully elucidated. Different proteoforms of αSyn have been observed not only in the central nervous system (CNS) but also in peripheral tissues and biofluids such as cerebrospinal fluid, plasma, saliva, olfactory mucosa, skin, salivary glands, retina, adrenal medulla, heart, and gastrointestinal tract in PD cases. Interestingly, the possibility to detect and quantify αSyn conformers in biofluids and in tissue biopsies reflects on the multisystem nature of the disorder and represents a promising diagnostic approach at the preclinical stage of PD, when neurodegeneration commences. Randomized controlled trials, cohort and cross-sectional studies and the validation of PD-specific screening questionnaires together with non-invasive and painless sample collection procedures and sophisticated technology for αSyn measurement can contribute to the achievement of timely diagnosis, monitoring and management of PD.

Carrying out robust clinical trials in large populations with the careful application of objective biomarkers and assessment of confounding factors will be essential to consolidate the therapeutic potentials of antioxidants for PD.
